# Comparison of Indices of Carbohydrate Quality and Food Sources of Dietary Fiber on Longitudinal Changes in Waist Circumference in the Framingham Offspring Cohort

**DOI:** 10.3390/nu13030997

**Published:** 2021-03-19

**Authors:** Caleigh M. Sawicki, Alice H. Lichtenstein, Gail T. Rogers, Paul F. Jacques, Jiantao Ma, Edward Saltzman, Nicola M. McKeown

**Affiliations:** 1 Jean Mayer USDA Human Nutrition Research Center on Aging at Tufts University, Boston, MA 02111, USA; Caleigh.Sawicki@tufts.edu (C.M.S.); Alice.Lichtenstein@tufts.edu (A.H.L.); Gail.Rogers@tufts.edu (G.T.R.); Paul.Jacques@tufts.edu (P.F.J.); 2 Gerald J. and Dorothy R. Friedman School of Nutrition Science and Policy, Tufts University, Boston, MA 02111, USA; Jiantao.Ma@tufts.edu (J.M.); Edward.Saltzman@tufts.edu (E.S.)

**Keywords:** carbohydrate quality, dietary fiber, abdominal adiposity, waist circumference, Framingham Offspring

## Abstract

The long-term impact of carbohydrate quality on abdominal weight gain is not fully understood. We aimed to examine the prospective relation of a carbohydrate quality index (CQI; defined by four criteria: dietary fiber, glycemic index, whole grain-to-total grain ratio, and solid-to-total carbohydrate ratio), total, cereal grain, vegetable, and fruit fiber, carbohydrate-to-total fiber ratio, and carbohydrate-to-cereal fiber ratio with changes in waist circumference (WC). Subjects were middle-aged to older, mostly white, participants in the Framingham Offspring cohort (*n* = 3101 subjects), with mean baseline age 54.9 ± 0.2 years (mean ± SE) and body mass index (BMI) 27.2 ± 0.1 kg/m^2^. Food frequency questionnaire (FFQ), health, and lifestyle data were collected approximately every four years over a median total follow-up of 18 years. Repeated measure mixed models were used to estimate adjusted mean change in WC per four-year interval across quartiles of carbohydrate variables. In the most adjusted model, a higher CQI was marginally associated with a smaller increase in WC (2.0 ± 0.1 vs. 2.4 ± 0.1 cm in highest vs. lowest quartile, *p*-trend = 0.04). Higher ratios of carbohydrate-to-fiber and carbohydrate-to-cereal fiber were associated with greater increases in WC per four-year interval (2.6 ± 0.1 vs. 2.0 ± 0.1 cm, *p*-trend < 0.001, and 2.5 ± 0.1 vs. 2.1 ± 0.1 cm in highest versus lowest categories, *p*-trend = 0.007, respectively); whereas higher intake of total fiber (1.8 ± 0.1 vs. 2.7 ± 0.1 cm, *p*-trend < 0.001), cereal fiber (2.0 ± 0.1 vs. 2.5 ± 0.1 cm, *p*-trend = 0.001), and fruit fiber (2.0 ± 0.1 vs. 2.7 ± 0.1 cm, *p*-trend < 0.001) were associated with smaller increases in WC compared to lower intakes. There was a significant interaction between total fiber and total carbohydrate (as % of total energy intake). After stratification, the association between fiber intake and change in WC was not maintained in the context of a high carbohydrate diet. Better carbohydrate quality, primarily higher fiber intake and lower carbohydrate-to-fiber ratios, may help attenuate increases in abdominal adiposity over time.

## 1. Introduction

Over the past few decades, the prevalence of abdominal obesity, primarily measured by waist circumference (WC), has increased in adults [1,2]. Notably, abdominal obesity is expected to continue increasing at a rate greater than overall obesity (measured by BMI) alone [3,4]. Excess abdominal adiposity, independent of overall adiposity, is associated with higher risk of metabolic disease [5,6], type 2 diabetes (T2D) [7,8,9], and cardiovascular disease (CVD) [7,10,11,12]. The relation between abdominal obesity and these diseases may be mediated through increased inflammation, dyslipidemia, and insulin resistance [5,13,14,15]. Lifestyle behaviors, such as poor diet and physical inactivity, are major modifiable factors contributing to excess body weight gain [16]. Yet to be resolved is the contribution of dietary carbohydrate quality to the development of adiposity, particularly abdominal adiposity.

About 50% of all calories consumed in the U.S. come from carbohydrates, and although some small improvements have been observed recently, this largely consists of low-quality carbohydrates from refined grains and added sugars [17]. Therefore, carbohydrates are often labeled as an underlying contributor to weight gain or metabolic disorders, with little emphasis placed on the dietary sources of carbohydrates. Observational studies have found measures of adiposity are lower among adults who consume more whole grain foods [18,19], dietary fiber [20,21], and fiber from cereal grains (also referred to as cereal fiber) [19,22,23]. In contrast, adiposity is higher among adults who consume diets rich in added sugar, primarily through sugar-sweetened beverages (SSBs) [24,25], and refined grains [21,24]. Some of these attributes of carbohydrates, such as whole grain or total fiber, have been used as surrogate markers of overall carbohydrate quality, which a growing body of evidence shows is a more important determinant of health than carbohydrate quantity alone [26,27,28,29,30,31].

Alternate approaches to those traditionally used to define carbohydrate quality include ratios such as total carbohydrate-to-total fiber (carb-to-fiber) and total carbohydrate-to-cereal fiber (carb-to-cereal fiber). These ratios have been associated with higher risk of T2D [28], CVD [29], and metabolic syndrome [32]. Another emerging method is the use of a multi-component carbohydrate quality index (CQI), which includes four attributes of carbohydrate quality: dietary fiber, glycemic index (GI), ratio of whole grain to total grain (whole plus refined grain), and ratio of solid to total carbohydrate. A diet that has a higher CQI (reflecting better carbohydrate quality), has been associated in observational studies with lower risk of obesity [33] and CVD [34]. More recently, Martinez-Gonzalez and colleagues found that increasing CQI was associated with improvements in CVD risk factors including lower body weight and WC after six months among adults with overweight/obesity and metabolic syndrome enrolled in the PREDIMED-Plus (Prevención Con Dieta Mediterránea Plus) lifestyle intervention trial [35]. However, a comparison of CQI, carbohydrate ratios, and other carbohydrate quality indices with long-term changes in abdominal adiposity in a large prospective cohort has yet to be examined. Therefore, the aim of the present study was to distinguish the relationship between new metrics of carbohydrate quality, as well as more traditional metrics, such as amount of fiber from different sources in relation to changes in waist circumference over time, in order to help inform public health guidelines.

## 2. Materials and Methods

Participants were enrolled in the National Heart, Lung, and Blood Institute (NHLBI) Framingham Heart Study (FHS) Offspring Cohort. The Offspring cohort began in 1971 with the recruitment of 5124 adult offspring (and their spouses) of the original FHS cohort. Follow-up visits occur approximately every four years and include standardized physical examinations, laboratory tests, and health- related questionnaires. Dietary assessment began at the fifth examination cycle, and therefore we used data beginning with the fifth exam through the ninth (most recently available) exam [5th (1991–1995, *n* = 3799;), 6th (1995–1998, *n* = 3532), 7th (1998–2001, *n* = 3539), 8th (2005–2008, *n* = 3021), and 9th (2011–2014, *n* = 2430)].

All FHS study protocols and procedures were conducted according to the guidelines of the Declaration of Helsinki and approved by the institutional review board for human research at Boston University. The current study was reviewed by the Tufts Health Sciences Institutional Review Board (#12822, approved 2/15/2018). All subjects provided their written informed consent for participation. 

### 2.1. Dietary Assessment

At each study exam, usual dietary intake over the previous year was assessed using the Harvard semi-quantitative Food Frequency Questionnaire (FFQ) [36]. The relative validity of this FFQ to capture both food and nutrient intake has been evaluated in several populations [36,37,38]. It includes a list of foods with standard serving sizes and 9 frequency categories, ranging from never or <1 serving per month to ≥6 servings per day. Dietary data was considered invalid if there were >12 food items left blank, if total energy intake was <600 kcal/d, or if total energy intake was >4000 kcal/d for females or >4200 kcal/d for males. Daily food group intake (including grains, fruits, vegetables, sugar-sweetened beverages and fruit juices, etc.) was calculated by multiplying the portion size of each food that was consumed by the consumption frequency and summing across all food items. Nutrient intakes (including fiber and carbohydrate) were calculated by multiplying the frequency of consumption of each food item by the nutrient content of the specified portion, according to the US Department of Agriculture food composition database and supplemented with other published sources [36]. Fiber intake from cereal grains (referred to as cereal fiber), fruits, and vegetables was calculated by summing the individual fiber contribution of each fruit and vegetable food item (see Supplementary Table S1 for the list of specific foods in each category). Fiber from legumes, nuts, and seeds was included in total fiber, but not assessed separately due to the relatively low consumption of these food items in this population (legumes and nuts/seeds contributed 5.8% and 2.6% of total dietary fiber at baseline, respectively). Isolated fiber supplements reported by subjects were included in total fiber intake. Determination of GI of specific food items and glycemic load have been described elsewhere [39,40]. A total dietary GI was then calculated by dividing the average daily glycemic load by the average daily carbohydrate intake [39].

The primary exposure of interest was the CQI, a summary score based on intake of dietary fiber (g/day), GI (negatively weighted), ratio of carbohydrates from whole grains (g/day) to carbohydrates from total cereal grains (whole plus refined grain) (g/day), and the ratio of solid carbohydrates (g/day) to total (liquid + solid) carbohydrates (g/day) [34]. Liquid carbohydrates consisted of SSB and fruit juices, and solid carbohydrates consisted of carbohydrates from all other food sources. Intakes were divided into quintile categories, and a score of 1–5 was assigned per quintile (score of 1 for the lowest quintile and 5 for the highest quintile for each component except for GI which was reverse scored, with lowest quintile receiving a score of 5 and highest a score of 1). Scores for all components were summed, giving the index a possible range of 4–20 with a higher score reflecting better carbohydrate quality (Table 1). All carbohydrate exposure variables were estimated as an average of values measured at the beginning and end of each exam interval (i.e., the average of two consecutive exams).

### 2.2. Waist Circumference

WC, the primary outcome of interest, was measured by a trained professional by applying anthropometric tape at the level of the umbilicus with the participant standing, at mid-respiration, breathing normally, and rounding to the nearest 0.25 inches. Body weight, a secondary outcome, was measured (to the nearest 0.5 lbs) on daily calibrated scales following standardized procedures. WC and weight measurements were converted to units of cm and kg, respectively, for analyses. Changes in outcomes were calculated as the change between consecutive exams. Since the actual time interval between exams could differ by subject, changes in outcomes were standardized by dividing the raw change by the number of years between exam dates and then expressing the result as four-year change (because exams are approximately four years apart, on average).

### 2.3. Covariates

Potential confounders of the relationship between carbohydrate quality and WC considered as covariates in analyses included: age (years); sex (male/female); current smoker (yes/no reported smoking regularly in the last year); physical activity (measured by the physical activity index (PAI), a score based on the sum of sedentary, light, moderate and vigorous metabolic equivalent task (MET) hours/week); alcohol consumption (g/day); pharmacological treatment of diabetes (if developed after baseline, yes/no); menopausal status (yes/no periods had stopped for ≥1 year); BMI (calculated as measured weight (kg) divided by height (m) squared (kg/m^2^)); and the percentage of total energy intake from saturated fatty acids (SFA). Since PAI was not available for exam 6, exam 5 values were carried forward. Age and sex were defined at baseline for each subject. BMI was captured at the start of each exam interval (also referred to as the periodic baseline), and all other covariates were estimated as an average of values measured at the beginning and end of each exam interval (i.e., the average of two consecutive exams), including yes/no variables, to account for potential changes in risk factors (e.g., if a participant reported smoking at exam 5, which was coded as 1, and reported not smoking by exam 6, which was coded 0, the value of 0.5 would be assigned to this variable in the model).

3789 subjects attended at least two exams between the 5th and 9th examination cycles. For the purpose of the present analysis, subjects were excluded if they had no valid dietary data (*n* = 71), no WC measurements (*n* = 8), did not have at least one consecutive follow-up exam (*n* = 366), or had diabetes at baseline (defined as non-fasting blood glucose ≥ 200 mg/dL, or fasting blood glucose ≥ 126 mg/dL or currently being treated for diabetes) (*n* = 240) (Supplementary Figure S1). Baseline varied by subject and was defined as the first exam for which there was consecutive follow-up data. Subjects contributed multiple observations if they had data from >2 consecutive exams. To minimize influence from those with the most extreme changes in WC, observations were excluded from final analyses if the four-year change in WC was not within 4 SDs of the mean four-year change, which led 3 additional subjects to be excluded. In total, 3101 subjects were included in final analyses, contributing 9053 exam-interval observations.

### 2.4. Statistical Analyses

All carbohydrate quality variables were adjusted for total energy intake using the residual method and categorized into quartiles [41]. Spearman correlation coefficients were used to evaluate the correlations between the CQI and all the other carbohydrate variables. Repeated measure mixed models with an unstructured covariance matrix were used to estimate adjusted mean four-year changes in WC per quartile category of each carbohydrate quality variable. A test for linear trend across quartiles was performed by taking the median value of each quartile and treating as a continuous variable. We adjusted for groups of covariates sequentially. Model 1 adjusted for age, sex, total energy intake, and the periodic baseline WC value. Model 2 adjusted for everything in model 1 plus smoking status, physical activity, alcohol consumption, menopausal status, medication use for diabetes, and SFA(%kcal/d). Models for the subtypes of fiber mutually adjusted for the other two subtypes of fiber. We additionally adjusted BMI at each exam interval (also called the periodic baseline BMI) separately in model 3. Sensitivity analyses substituting SFA (%kcal/d) for total fat (%kcal/d) or the ratio of polyunsaturated to saturated fatty acids led to similar results (data not shown). Analysis for change in weight was conducted in a similar manner as a secondary outcome of interest. Effect modification by sex, age, BMI, and carbohydrate intake (as percent of total energy) was tested by including the cross-product term with each carbohydrate quality variable in the corresponding models and assessing the statistical significance of the likelihood ratios for change in WC. When significant interactions were detected, stratified analysis was performed. All analyses were conducted in SAS 9.4 (SAS Institute, Cary, NC, USA). All statistical tests were two-sided, and a *p*-value of <0.05 was considered statistically significant. Since tests for effect modification were exploratory, these were considered statistically significant at a Bonferroni-corrected *p* < 0.0125 (0.05/4 interaction tests).

## 3. Results

In total, 3101 subjects met the criteria for inclusion in final analyses. Over the median total follow-up time of 18.1 years (IQR = 7.2), an average of 4 exams were attended (of a possible 5), contributing a total of 9053 exam-interval observations. Subjects had a mean baseline age of 54.9 ± 0.2 years (mean ± SE), BMI of 27.2 ± 0.1 kg/m^2^, and 54.3% were females (Table 2). Mean baseline WC was 92.6 ± 0.23 cm (87.3 ± 0.34 and 99.0 ± 0.28 cm for females and males, respectively). On average, carbohydrates constituted 51% of total energy intake. Dietary fiber intake at baseline was 18.2 ± 8.0 g/day, of which cereal fiber was the top contributor (29.1% of total fiber), followed by vegetable fiber (24.2%) and fruit fiber (18.6%). Those in the highest quartile of CQI were less likely to be smokers, tended to have the highest total energy, total fiber, whole grain and vegetable intakes, and lowest refined grain and SSB intakes (baseline characteristics across quartiles of the other carbohydrate variables of interest are presented in Supplementary Table S2). The average four-year change in WC was an increase of 2.7 ± 6.3 cm but ranged from a decrease of 24.3 cm to an increase of 29.6 cm. Consideration of sex as a potential effect modifier did not alter the association between dietary exposures and four-year change in WC. Therefore, females and males were combined in the subsequent analyses.

The CQI was significantly correlated with each of its component parts: GI (Spearman *r* = −0.55), total dietary fiber (*r* = 0.62), whole grain-to-total grain ratio (*r* = 0.56), and solid-to-total carbohydrate ratio (*r* = 0.60) (*p* < 0.0001 for each, Table 3). CQI was also correlated with the other measures of carbohydrate quality (*p* < 0.0001, Table 4), having correlation coefficients ranging from 0.07 with total carbohydrate to −0.69 with carb-to-fiber ratio.

No statistically significant trend was observed across increasing quartiles of the CQI in relation to four-year change in WC in the first two models (Table 5). However, after adjusting for BMI, we observed a marginally significant relationship, where a higher CQI was associated with a smaller increase in WC (2.0 ± 0.1 vs. 2.4 ± 0.1 in highest vs. lowest quartile, *p*-trend = 0.04). We next examined the association between each individual component of the CQI and change in WC (Supplementary Table S3). Higher GI was associated with greater increase in WC (2.5 ± 0.1 vs. 1.7 ± 0.1 cm in highest vs. lowest quartile, *p*-trend < 0.001), whereas higher total fiber and whole grain-to-total grain ratio were associated with smaller increases in WC (1.7 ± 0.1 vs. 2.5 ± 0.1 cm in highest vs. lowest quartile, *p*-trend < 0.001, and 1.5 ± 0.1 vs. 3.1 ± 0.1 cm, *p*-trend < 0.001, respectively). These associations remained significant after further adjustment for BMI. The association of solid-to-total carbohydrate ratio and change in WC was not statistically significant.

A higher ratio of carb-to- fiber was associated with slightly greater four-year increase in WC (2.3 ± 0.1 vs. 1.9 ± 0.1 cm in highest vs. lowest quartile, *p*-trend = 0.007, Table 5), and this association remained after further adjusting for BMI. A similar positive association was observed between higher carb-to-cereal fiber ratio and greater WC (2.3 ± 0.1 vs. 1.9 ± 0.1 cm, *p*-trend = 0.003), and remained significant after further adjustment for BMI.

No significant association was observed between intake of total carbohydrate (g/d) and four-year change in WC. However, higher intake of total fiber (g/d), cereal fiber (g/d), and fruit fiber (g/d) were associated with a smaller increase in WC (1.7 ± 0.1 vs. 2.5 ± 0.1 cm in highest vs. lowest quartile, *p*-trend < 0.001, 1.8 ± 0.1 vs. 2.4 ± 0.1 cm *p*-trend < 0.001, and 1.9 ± 0.1 vs. 2.4 ± 0.1 cm, *p*-trend = 0.014, respectively, Table 6), and these associations remained significant after further adjustment for BMI. No significant association between vegetable fiber with change in WC was observed.

**Figure 1:**
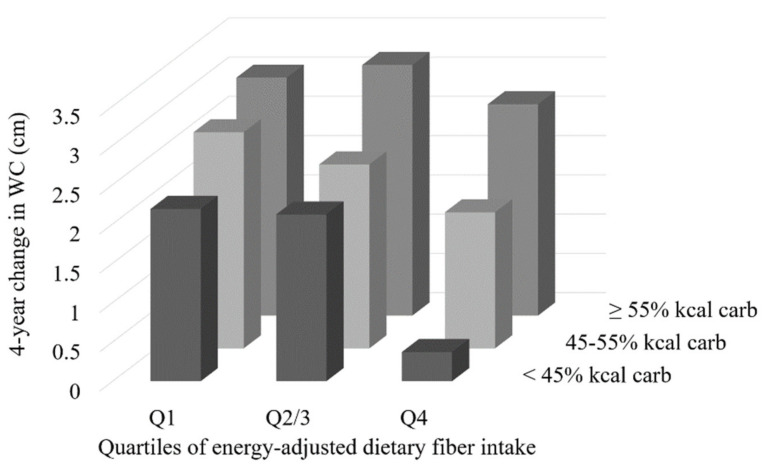
Combined association of total fiber and percent of energy from carbohydrate on mean four-year change in waist circumference (cm) in 3101 participants of the Framingham Cohort Study; Adjusted for age, sex, energy intake, periodic baseline WC, smoking status, physical activity score, alcohol, menopausal status, medication for diabetes, SFA (%kcal/d), and periodic baseline BMI; second and third quartile of fiber intake have been collapsed for visualization; BMI (body mass index); SFA (saturated fatty acids); WC (waist circumference).

Effect modification by carbohydrate intake (as percent of total energy) was observed in the association between total dietary fiber and change in WC (*p* = 0.009 for the interaction term). Subsequent stratification by categories of carbohydrate intake (<45%, 45% to <55%, or ≥55% of total energy from carbohydrate) revealed that the association between higher fiber intake and smaller increase in WC is not maintained in the context of a high carbohydrate diet (Table 7). Further, when carbohydrate intake is <45% of total energy intake, the observed effect of dietary fiber on maintaining WC is enhanced (in fully adjusted models, those in the highest quartile of fiber intake had 1.8 cm smaller increase in WC compared to those in the lowest quartile, whereas the difference between quartiles was only 0.9 cm in the non-stratified data). Figure 1 depicts the combined effects of total carbohydrate and total fiber intake (Q2 and Q3 were collapsed for ease of visualization).

We also detected a significant interaction between total carbohydrate intake and BMI (*p* < 0.001), but stratification by BMI did not reveal any statistically significant associations with WC (data not shown). No statistically significant associations were observed between any of the carbohydrate quality measures and overall body weight (Supplementary Table S4).

## 4. Discussion

In a large, community-based prospective cohort of US adults, the intake of a lower quality carbohydrate diet, as defined by higher carb-to-fiber and carb-to-cerealfiber ratios, was associated with a greater increase in WC over time. Consistent with these findings, total fiber and fiber from cereal grain and fruit sources were associated with better maintenance (i.e., smaller increases) of WC over time. These associations were independent of BMI and several demographic and lifestyle factors. Interestingly, although both total fiber and whole grain (a predominant source of cereal fiber) are two components of the CQI, we did not observe a strong association between CQI and change in WC. Although the CQI accounts for multiple aspects of carbohydrate nutrition, simpler measures such as the carb-to-fiber and carb-to-cereal fiber ratios, which correlated well with the CQI, were better predictors of change in WC.

Although the ‘optimal’ ratio of carbohydrate, fat, and protein remains an area of debate [42], as with dietary fat, there has been increased focus on carbohydrate quality over quantity in determining risk of chronic disease. The CQI is a relatively new index for measuring carbohydrate quality and provides a multidimensional approach to defining carbohydrate quality. Higher CQI has been associated with a lower risk of nutritional inadequacy (in terms of micronutrient requirements) [43,44], lower risk of incident CVD [34], and lower prevalence and incidence of obesity [33,45]. Of note, in the same study that found an association between higher CQI and lower incidence of obesity, no association was found between CQI and weight gain [33]. Our results support these findings. By contrast, in a recent 12-month weight loss and intervention trial, improvement in CQI, compared to those who had a reduction in CQI, was associated with improvement in several CVD risk factors, including decreased weight and WC (−1.5 kg and −1.5 cm in highest compared to lowest quintile) [35]. We observed a marginally significant association between CQI and four-year change in WC. One important difference between the two studies is that we were looking at average CQI over time, while the previous study was measuring change in CQI.

It is possible that the CQI could be improved as evidence in the field of carbohydrate quality evolves. For example, there is some evidence that SSBs and fruit juices may have differential metabolic effects [46,47,48] and, therefore, should not necessarily be grouped together in the calculation of liquid carbohydrates. Recent evidence has cast doubt on the reliability and utility of GI, particularly as it is being promoted for use by nondiabetic individuals [49]. Using glycemic load, which accounts for the amount of carbohydrate consumed, in place of GI, or removing this component of the CQI altogether should be explored. Given the interaction we detected with total carbohydrate intake, this may also be important to incorporate into the index.

The ratios of carb-to-fiber or carb-to-cereal fiber are alternate indices to the CQI used to define carbohydrate quality. Higher ratios, reflective of a poorer carbohydrate quality diet, have been associated with metabolic syndrome and associated risk factors [32], as well as higher risk of CHD [29] and T2D [28]. Both ratios appeared to be similarly associated with better maintenance of WC over time. We also found evidence of effect modification by total carbohydrate intake (as a percent of total energy) in the association between total fiber intake and change in WC. When combined, the effect of higher fiber and lower total carbohydrate (<45% total calories) intake resulted in better maintenance of WC over time, similar to what we observed directly with the carb-to-fiber ratio. This observation is also similar to observations by McKeown et al. who found that higher intake of whole grain was associated with lower abdominal adipose tissue only in the context of low refined grain intake [50]. Although both ratios were similar in terms of the observed associations with WC, the carb-to-fiber ratio is more easily translatable to the general public. A cut-off of 10:1 for the ratio of carbohydrate to fiber has been previously shown to be effective in identifying higher quality carbohydrate or whole grain food items [51,52]. Our data also support the concept that total carbohydrate intake alone is not a good predictor of change in WC, contrary to what many popular diets promote.

The observation that total fiber and subtypes of fiber (mostly cereal fiber) are associated with better maintenance of WC are consistent with other studies [19,20,23] and our previous findings of an association between higher whole grain intake and smaller four-year increase in WC in the same cohort (data not yet published). We also observed that higher intake of fruit fiber was associated with smaller increases in WC, whereas other studies have reported no significant association between fruit fiber and WC [19,20]. However, a systematic literature review concluded higher fiber and fruit intake, but not vegetable intake, was associated with smaller increase in WC, [53]. Additionally, a prospective study of male US health professionals aged 40–75 also observed significant inverse associations of weight change with changes in cereal fiber and fruit fiber, but not vegetable fiber [54]. In contrast, in our cohort, there was no significant association between overall body weight and measures of dietary fiber intake.

Although the effect sizes we observed were not large (the differences in four-year WC change between highest and lowest quartiles ranged from 0.4 to 1.8 cm), small gains in abdominal adiposity accumulate over time and can have an impact on disease risk. For example, in a meta-regression analysis of 15 prospective studies, just a 1 cm increase in WC increased the risk of CVD events by 2% [55]. Based on a more recent publication of 12,337 middle-aged and elderly adults, males and females who gained >5 cm in WC over 16 years had 50% and 25% higher risk of total mortality, respectively, and males had 84% higher risk of CVD mortality [56]. In the present study with a median follow-up period of 18 years, being in the highest vs. lowest quartile of carbohydrate quality contributed up to an average of 1.8 to 8.3 cm less increase in WC gain.

The proposed mechanisms whereby high fiber may be associated with better maintenance of WC include enhancing satiety, slowing glucose absorption rate and dampening of subsequent insulin response, and/or providing fermentable material for microbiota, producing short-chain fatty acids. The latter has been reported to trigger the release of gastrointestinal satiety hormones and accelerate the rate of lipid oxidation and lipolysis [57,58,59,60]. In addition, diets high in poor quality carbohydrates are often characterized by high sugar content and GI, which have been linked to inflammation, insulin resistance, and fat accumulation [60].

Limitations of the present study include generalizability. The Framingham Offspring cohort is a relatively homogenous cohort of middle-aged to older, white Americans. However, they were also at higher risk of abdominal obesity, defined as a WC of >88 cm and >102 cm for females and males, respectively. The average baseline WC was 87.3 ± 0.34 and 99.0 ± 0.28 cm for females and males, respectively. The dietary data was collected using an FFQ, which can be subject to recall and self-report biases. However, FFQs are widely used and are appropriate for estimating high or low consumers of specific foods or nutrients relative to one another [61]. The foods reported on the FFQ were matched to a comprehensive nutrient database to estimate nutrient composition. Although we controlled for several potential confounding variables, we cannot rule out the possibility of residual confounding by other lifestyle factors influencing our results. A major strength of this study is the large prospective nature of the FHS cohort with a high participation rate of follow-up. We were able to utilize data from repeated measures of both the exposures and outcomes over a median of 18 years of total follow-up. Additionally, our statistical approach allowed us to account for potential changes in diet over time rather than assuming a consistent level of intake for each of our exposures over the long follow-up period [62].

## 5. Conclusions

It is estimated that by 2030, nearly 56% of males and 80% of females will be abdominally obese [2], which predisposes individuals to metabolic abnormalities, including dyslipidemia, insulin resistance, inflammation, and hypertension. Our findings suggest that a focus on better carbohydrate quality defined by a higher CQI and lower carb-to-fiber ratio, with a particular focus on cereal and fruit fiber, may be an important dietary modification to attenuate age-related increases in waist circumference, a measure of abdominal adiposity. Although the multi-component CQI was marginally associated with lower WC and deserves further investigation or improvement, the simpler ratios of carb-to-fiber or carb-to-cereal fiber may be more useful measures.

## Figures and Tables

**Table 1:** Criteria used to calculate carbohydrate quality index (CQI).

Components of CQI	Score Range	Criteria for Minimum Score	Criteria for Maximum Score
Glycemic index	1–5	Highest quintile of glycemic index	Lowest quintile of glycemic index
Dietary fiber intake (g/d)	1–5	Lowest quintile of dietary fiber intake	Highest quintile of dietary fiber intake
Ratio of whole grain/total grain	1–5	Lowest quintile of the ratio	Highest quintile of the ratio
Ratio of solid carbohydrates/total (solid + liquid) carbohydrates	1–5	Lowest quintile of the ratio	Highest quintile of the ratio
Total index	4–20		

**Table 2:** Baseline characteristics across categories of CQI score among 3101 participants of the Framingham Cohort Study (mean (SE) or percentage).

		Baseline CQI Score			
	Total	Q 14–9	Q 210–12	Q3 13–14	Q4 15–20
Characteristics ^1^					
*n*	3101	701	1009	620	771
Age	54.9 (0.17)	54.2 (0.36)	54.6 (0.3)	55.1 (0.38)	55.9 (0.34)
Sex (%M)	45.7	51.5	49.5	44.0	36.9
Weight (kg)	76.9 (0.25)	77.0 (0.52)	77.0 (0.44)	76.5 (0.56)	77.0 (0.50)
WC (cm)	92.6 (0.23)	93.5 (0.48)	92.7 (0.40)	91.5 (0.51)	92.7 (0.46)
BMI (kg/m^2^)	27.2 (0.08)	27.3 (0.18)	27.2 (0.15)	27.0 (0.19)	27.1 (0.17)
PAI score	35.0 (0.11)	35.1 (0.23)	34.8 (0.20)	34.9 (0.25)	35.0 (0.23)
Current smoker (%)	18.0	25.8	21.4	13.8	10.0
Hypertension (%)	46.6	49.3	47.7	44.8	44.2
BP medication (%)	17.8	20.8	16.3	17.3	17.3
Lipid medication (%)	7.6	9.5	6.9	7.1	7.3
Menopausal (%)	36.2	36.9	35.6	35.6	36.6
Dietary Intakes ^2^					
Total energy (kcal/d)	1870 (10.8)	1728 (22.3)	1813 (18.6)	1907 (23.7)	2045 (21.3)
Carbohydrate (% kcal/d)	50.9 (0.20)	51.6 (0.30)	49.8 (0.30)	51.0 (0.30)	51.9 (0.30)
Fat (% kcal/d)	29.9 (0.11)	30.2 (0.24)	30.7 (0.20)	29.7 (0.25)	28.8 (0.23)
Protein (% kcal/d)	16.8 (0.06)	15.4 (0.12)	16.8 (0.1)	17.1 (0.13)	17.8 (0.11)
Total carbohydrate (g/d)	238.9 (0.75)	242.4 (1.59)	235.1 (1.32)	237.8 (1.68)	241.7 (1.53)
CQI score	12.1 (0.06)	7.5 (0.04)	11.1 (0.03)	13.5 (0.04)	16.4 (0.04)
Total fiber (g/d)	18.2 (0.11)	13.8 (0.19)	16.7 (0.15)	19.2 (0.2)	23.2 (0.18)
Whole grain (svg/d)	1.0 (0.02)	0.4 (0.03)	0.8 (0.03)	1.1 (0.03)	1.7 (0.03)
Refined grain (svg/d)	3.0 (0.03)	3.5 (0.05)	3.4 (0.05)	3.1 (0.06)	2.5 (0.05)
Fruit (svg/d)	2.1 (0.03)	1.8 (0.05)	2.0 (0.04)	2.2 (0.06)	2.6 (0.05)
Vegetables (svg/d)	2.8 (0.03)	2.1 (0.06)	2.7 (0.05)	3.1 (0.06)	3.9 (0.06)
SSBs (svg/d)	1.3 (0.02)	2.1 (0.04)	1.3 (0.03)	1.0 (0.04)	0.6 (0.04)
Total alcohol (g/d)	10.9 (0.28)	10.8 (0.59)	10.9 (0.49)	11.3 (0.62)	10.8 (0.57)
SFA (% kcal/d)	10.4 (0.05)	10.8 (0.11)	10.7 (0.09)	10.2 (0.11)	9.7 (0.10)

^1^ Adjusted for age and sex; ^2^ Adjusted for age, sex, and total energy intake; BMI (body mass index); CQI (carbohydrate quality index); PAI (physical activity index); SFA (saturated fatty acid); SSB (sugar-sweetened beverage); WC (waist circumference).

**Table 3:** Spearman correlation coefficients between CQI and its component factors.

	GI	Total Fiber	Whole Grain: Total Grain	Solid: Total Carbohydrate
CQI				
*r*	−0.55	0.62	0.56	0.60
*p*-value	<0.0001	<0.0001	<0.0001	<0.0001
GI				
*r*		−0.10	−0.22	−0.32
*p*-value		<0.0001	<0.0001	<0.0001
Total Fiber				
*r*			0.38	0.16
*p*-value			<0.0001	<0.0001
Whole grain: total grain				
*r*				0.20
*p*-value				<0.0001

CQI (carbohydrate quality index); GI (glycemic index).

**Table 4:** Spearman correlation coefficients between energy-adjusted carbohydrate quality variables.

	Carbohydrate	Total Fiber	Cereal Fiber	Vegetable Fiber	Fruit Fiber	Carbohydrate: Total Fiber	Carbohydrate: Cereal Fiber
CQI							
*r*	0.07	0.67	0.40	0.50	0.47	−0.69	−0.39
*p*-value	<0.0001	<0.0001	<0.0001	<0.0001	<0.0001	<0.0001	<0.0001
Carbohydrate							
*r*		0.44	0.38	0.11	0.47	0.11	−0.05
*p*-value		<0.0001	<0.0001	<0.0001	<0.0001	<0.0001	<0.0001
Total fiber							
*r*			0.59	0.67	0.71	−0.79	−0.45
*p*-value			<0.0001	<0.0001	<0.0001	<0.0001	<0.0001
Cereal fiber							
r				0.15	0.26	−0.39	−0.90
*p*-value				<0.0001	<0.0001	<0.0001	<0.0001
Vegetable fiber							
*r*					0.39	−0.67	−0.11
*p*-value					<0.0001	<0.0001	<0.0001
Fruit fiber							
*r*						−0.47	−0.10
*p*-value						<0.0001	<0.0001
Carbohydrate: total fiber							
*r*							0.46
*p*-value							<0.0001

CQI (carbohydrate quality index).

**Table 5:** Means (SE) of four-year change in waist circumference (cm) by quartiles of different carbohydrate quality metrics in 3101 participants of the Framingham Cohort Study.

	Energy-Adjusted Quartiles				
	Q1	Q2	Q3	Q4	*p*-Trend
CQI ^1^					
*n* (observations)	2263	2263	2264	2263	
Median (range)	8.5 (3.7–9.9)	11.0 (9.9–12.0)	13.0 (12.0–14.0)	15.5 (14.0–21.2)	
Model 1	2.11 (0.10)	2.28 (0.10)	2.31 (0.10)	1.84 (0.10)	0.08
Model 2	2.11 (0.10)	2.29 (0.10)	2.32 (0.10)	1.88 (0.10)	0.15
Model 3	2.39 (0.11)	2.44 (0.11)	2.47 (0.11)	2.04 (0.11)	0.04
Carbohydrate: total fiber					
*n*	2263	2263	2264	2263	
Median (range)	9.6 (4.2–10.6)	11.5 (10.6–12.3)	13.3 (12.3–14.5)	16.5 (14.5–55.0)	
Model 1	1.84 (0.09)	2.16 (0.10)	2.33 (0.11)	2.26 (0.11)	0.003
Model 2	1.87 (0.10)	2.19 (0.10)	2.32 (0.11)	2.27 (0.11)	0.007
Model 3	2.02 (0.10)	2.32 (0.11)	2.48 (0.11)	2.58 (0.12)	<0.001
Carbohydrate: cereal fiber					
*n*	2263	2263	2264	2263	
Median (range)	29.7 (9.2–35.3)	40.4 (35.3–45.6)	51.6 (45.6–60.0)	75.3 (60.0–7885.1)	
Model 1	1.90 (0.09)	2.03 (0.10)	2.37 (0.11)	2.28 (0.11)	0.003
Model 2	1.91 (0.10)	2.04 (0.10)	2.39 (0.11)	2.31 (0.11)	0.003
Model 3	2.10 (0.11)	2.22 (0.11)	2.56 (0.11)	2.49 (0.12)	0.007

^1^ Because the CQI score was adjusted for energy, the range of possible scores is no longer exactly 4–20 as described in the methods; Model 1: periodic baseline age, sex, energy, periodic baseline waist circumference; Model 2: Model 1 + current smoker (y/n), physical activity score, alcohol (g/d), menopausal status, medication use for diabetes, SFA (%kcal/d); Model 3: Model 2 + periodic baseline BMI; BMI (body mass index); CQI (carbohydrate quality index); SFA (saturated fatty acid).

**Table 6:** Means (SE) of four-year change in waist circumference (cm) by quartiles of total carbohydrate, dietary fiber, and different food sources of fiber in 3101 participants of the Framingham Cohort Study.

	Energy-Adjusted Quartiles				
	Q1	Q2	Q3	Q4	*p*-Trend
Total carbohydrate					
*n* (observations)	2263	2263	2264	2263	
Median (range)	180 (36–197)	210 (197–220)	230 (220–242)	258 (242–366)	
Model 1	2.14 (0.10)	2.09 (0.10)	2.07 (0.10)	2.22 (0.11)	0.70
Model 2	2.05 (0.13)	2.08 (0.10)	2.12 (0.11)	2.34 (0.13)	0.19
Model 3	2.13 (0.14)	2.26 (0.11)	2.37 (0.11)	2.59 (0.14)	0.05
Total fiber					
*n*	2263	2263	2264	2263	
Median (range)	12.8 (5.6–14.7)	16.2 (14.7–17.6)	19.2 (17.6–21.1)	23.9 (21.1–54.1)	
Model 1	2.40 (0.10)	2.24 (0.10)	2.16 (0.10)	1.75 (0.10)	<0.001
Model 2	2.51 (0.12)	2.29 (0.11)	2.16 (0.10)	1.68 (0.11)	<0.001
Model 3	2.72 (0.13)	2.53 (0.11)	2.34 (0.11)	1.80 (0.12)	<0.001
Cereal fiber					
*n*	2263	2263	2264	2263	
Median (range)	3.0 (0.4–3.8)	4.4 (3.8–5.0)	5.7 (5.0–6.7)	8.0 (6.7–33.8)	
Model 1	2.30 (0.11)	2.32 (0.10)	2.13 (0.10)	1.81 (0.10)	<0.001
Model 2	2.38 (0.11)	2.36 (0.11)	2.14 (0.10)	1.79 (0.10)	<0.001
Model 3	2.54 (0.12)	2.53 (0.11)	2.32 (0.11)	2.02 (0.11)	0.001
Vegetable fiber					
*n*	2263	2263	2264	2263	
Median (range)	2.3 (0.0–2.9)	3.5 (2.9–4.0)	4.7 (4.0–5.4)	6.7 (5.4–27.6)	
Model 1	2.16 (0.10)	2.15 (0.10)	2.13 (0.10)	2.07 (0.10)	0.498
Model 2	2.11 (0.11)	2.18 (0.10)	2.20 (0.10)	2.18 (0.10)	0.686
Model 3	2.34 (0.12)	2.40 (0.11)	2.40 (0.11)	2.27 (0.11)	0.584
Fruit fiber					
*n*	2263	2263	2264	2263	
Median (range)	1.2 (0.0–1.9)	2.6 (1.9–3.3)	4.0 (3.3–4.9)	6.3 (4.9–24.7)	
Model 1	2.36 (0.10)	2.10 (0.10)	2.16 (0.10)	1.88 (0.10)	0.002
Model 2	2.38 (0.11)	2.14 (0.10)	2.21 (0.10)	1.93 (0.11)	0.014
Model 3	2.68 (0.12)	2.4 (0.11)	2.34 (0.11)	1.98 (0.12)	<0.001

Model 1: periodic baseline age, sex, energy, periodic baseline waist circumference; Model 2: Model 1 + current smoker (y/n), physical activity score, alcohol (g/day), menopausal status, medication use for diabetes, SFA (%kcal/d); models for subtypes of fiber are mutually adjusted for the other two subtypes of fiber; Model 3: Model 2 + periodic baseline BMI; BMI (body mass index); CQI (carbohydrate quality index); SFA (saturated fatty acid).

**Table 7:** Means (SE) of four-year change in waist circumference (cm) by quartiles of total fiber intake, stratified by totalcarbohydrate intake, in 3101 participants of the Framingham Cohort Study.

	Energy-Adjusted Quartiles of Fiber Intake				
	Q1	Q2	Q3	Q4	*p*-Trend
<45% E from carbohydrates					
*n*	1090	691	402	195	
Median (range)	12.6 (5.8–14.7)	16.1 (14.7–17.6)	19.0 (17.6–21.1)	23.4 (21.1–45.7)	
Model 1	2.19 (0.14)	2.01 (0.18)	1.76 (0.22)	0.27 (0.31)	<0.001
Model 2	2.20 (0.15)	2.04 (0.18)	1.80 (0.23)	0.31 (0.33)	<0.001
Model 3	2.20 (0.16)	2.20 (0.18)	1.97 (0.23)	0.36 (0.33)	<0.001
(45% to 55%) E from carbohydrates					
*n*	895	1214	1278	1040	
Median (range)	13.1 (6.3–14.7)	16.3 (14.7–17.6)	19.2 (17.6–21.1)	23.3 (21.1–46.8)	
Model 1	2.41 (0.18)	2.18 (0.15)	2.13 (0.14)	1.70 (0.15)	0.002
Model 2	2.49 (0.19)	2.20 (0.15)	2.16 (0.14)	1.64 (0.16)	0.001
Model 3	2.75 (0.20)	2.40 (0.16)	2.29 (0.15)	1.82 (0.17)	0.001
≥ 55% E from carbohydrates					
*n*	278	358	584	1028	
Median (range)	12.7 (5.6–14.7)	16.4 (14.7–17.6)	19.4 (17.6–21.1)	24.8 (21.1–54.1)	
Model 1	2.59 (0.35)	2.90 (0.31)	2.66 (0.23)	2.33 (0.17)	0.162
Model 2	2.36 (0.37)	2.85 (0.32)	2.61 (0.23)	2.48 (0.18)	0.778
Model 3	3.04 (0.38)	3.38 (0.32)	3.08 (0.24)	2.69 (0.19)	0.137

Model 1: periodic baseline age, sex, energy, periodic baseline waist circumference; Model 2: Model 1 + current smoker (y/n), physical activity score, alcohol (g/d), menopausal status, medication use for diabetes, SFA (%kcal/d); Model 3: Model 2 + periodic baseline BMI; BMI (body mass index); CQI (carbohydrate quality index); E (total energy intake); SFA (saturated fatty acid).

## Data Availability

Data described in the manuscript, codebook, and analytic code cannot be made available because the authors are prohibited from distributing or transferring the data and codebooks on which their research was based to any other individual or entity under the terms of an approved NHLBI Framingham Heart Study Research Proposal and Data and Materials Distribution Agreement through which the authors obtained these data.

## Supplementary Materials

The following are available online at https://www.mdpi.com/2072-6643/13/3/997/s1, Figure S1: Flow chart of included/excluded participants in analyses; Table S1: Food items contributing to each fiber subcategory; Table S2: Baseline characteristics across categories of carbohydrate quality variables among 3101 participants of the Framingham Cohort Study (mean (SE) or percentage); Table S3: Means (SE) of four-year change in waist circumference (cm) by quartiles of components of the CQI in 3101 participants of the Framingham Cohort Study; Table S4: Means (SE) of four-year change in body weight (kg) by quartiles of different carbohydrate quality metrics in 3101 participants of the Framingham Cohort Study.
