# Supplementary material for: Comparison of Indices of Carbohydrate Quality and Food Sources of Dietary Fiber on Longitudinal Changes in Waist Circumference in the Framingham Offspring Cohort
Source: Nutrients. 2021 Mar 19;13(3):997. doi: 10.3390/nu13030997 (PMC8003409; doi:10.3390/nu13030997)
Supplement: Supplementary file 1 [file nutrients-13-00997-s001.pdf]

Online Supporting Material

Table S1. Foods items contributing to each fiber subcategory

Food Group	Food items included
Cereal grains	Ready to eat breakfast cereal, cooked oatmeal, other cooked breakfast cereal, bread (white, dark, whole wheat/oatmeal/grain bread, rye/pumpernickel), English muffin/bagel/roll, muffin/biscuit, rice (white or brown), pasta, other grains, pancake/waffle, crackers, pizza, popcorn, tortillas, breakfast bar, energy bar, low carb bar, pretzels, sandwich, toast, granola bar, stuffing, corn muffin/cornbread, cheese curls, breadsticks, rice cake, polenta/grits, croutons, goldfish crackers, Chex party mix, matzoh, graham crackers, mixed dishes with grain components (soups, burritos, tacos, enchiladas)
Fruits	raisins, prunes, prune juice, bananas, cantaloupe, watermelon, apples/pears, apple juice/cider, oranges, orange juice, grapefruit, grapefruit juice, other fruit juice, strawberries, blueberries, peaches, apricots, plums, avocados, dates, dried apricots
Vegetables	tomato, tomato juice, tomato sauce, red chili sauce/salsa, string beans, broccoli, cabbage/cole slaw, cauliflower, brussel sprouts, carrots, corn, mixed vegetables, winter squash, summer squash, yams/sweet potatoes, spinach, kale/chard, lettuce, celery, beets, alfalfa sprouts, garlic, peppers, onions, ketchup, artichoke

Online Supporting Material

Figure S1. Flow chart of included/excluded participants in analyses

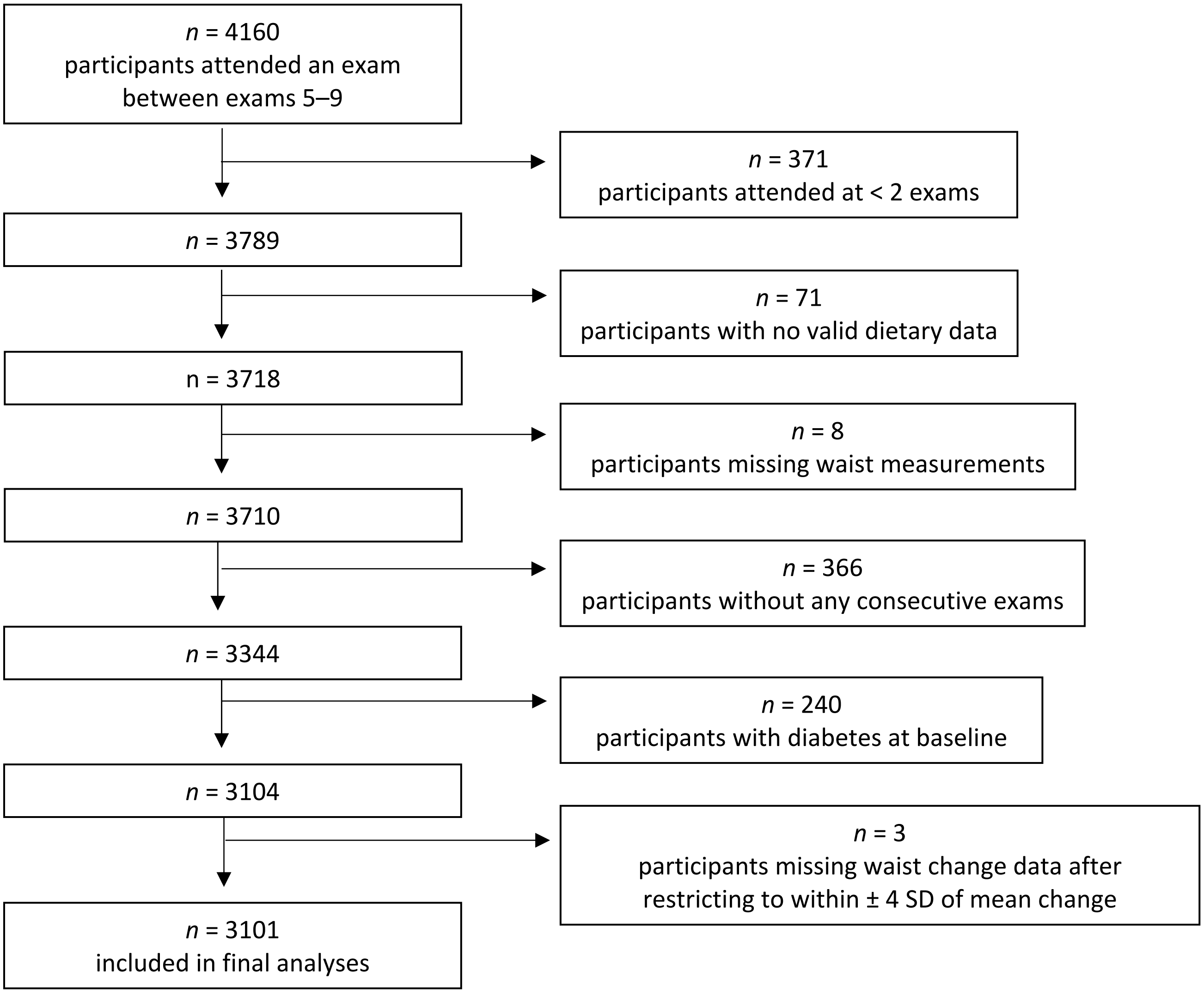

Online Supporting Material

Table S2. Baseline characteristics across categories of carbohydrate quality variables among 3101 participants of the Framingham Cohort Study (mean (SE) or percentage)

	Baseline carbohydrate-to-fiber ratio				Baseline carbohydrate-to-cereal fiber ratio			
	Q1<br>4.0-11.2	Q2<br>11.2-13.1	Q3<br>13.1-15.7	Q4<br>15.7-67.2	Q1<br>8.0-36.3	Q2<br>36.3-48.2	Q3<br>48.2-66.8	Q4<br>66.9-551.8
Characteristics[1]								
n	775	775	776	775	775	775	776	775
Age	57.1 (0.34)	55.4 (0.34)	54.8 (0.34)	52.3 (0.34)	55.5 (0.34)	55 (0.34)	54.6 (0.34)	54.6 (0.34)
Sex (%M)	33.0	42.7	49.1	58.1	42.5	43.1	45.4	51.9
Weight (kg)	76.6 (0.5)	77.1 (0.5)	77.5 (0.5)	76.5 (0.5)	76.2 (0.5)	77.3 (0.5)	77.7 (0.5)	76.4 (0.5)
WC (cm)	92.2 (0.46)	92.5 (0.45)	93.2 (0.45)	92.5 (0.46)	91.7 (0.45)	92.7 (0.45)	93.5 (0.45)	92.6 (0.45)
BMI (kg/m^2)	27 (0.17)	27.1 (0.17)	27.5 (0.17)	27.1 (0.17)	26.8 (0.17)	27.2 (0.17)	27.5 (0.17)	27.2 (0.17)
PAI score	35.1 (0.23)	35.0 (0.23)	34.7 (0.22)	35.0 (0.22)	34.6 (0.22)	34.8 (0.22)	35.2 (0.22)	35.2 (0.22)
Current smoker (%)	11.0	14.2	18.5	28.5	8.7	14.3	20.1	29.0
Hypertension (%)	43.2	49.2	45.5	48.5	44.6	45.9	48.9	47.1
BP medication (%)	17.5	19.1	18.6	15.9	17.0	17.4	20.5	16.2
Lipid medication (%)	6.9	8.3	8.4	6.8	8.1	8.3	7.9	6.1
Menopausal (%)	36.1	34.0	36.2	38.3	36.1	36.9	35.3	36.3
Dietary Intakes[2]								
Total energy (kcal/d)	1782 (21.7)	1845 (21.4)	1859 (21.4)	1996 (21.7)	1821 (21.5)	1899 (21.5)	1919 (21.5)	1842 (21.6)
Carbohydrate (% E)	50.8 (0.3)	50.0 (0.3)	50.3 (0.3)	52.7 (0.3)	52.1 (0.3)	50.5 (0.3)	50.0 (0.3)	51.2 (0.3)
Fat (% E)	28.7 (0.23)	30.2 (0.23)	30.6 (0.23)	30.2 (0.23)	28.5 (0.23)	30.2 (0.23)	31 (0.23)	29.9 (0.23)
Protein (% E)	18.4 (0.11)	17.2 (0.11)	16.6 (0.11)	15.1 (0.11)	17.7 (0.11)	17.3 (0.11)	16.7 (0.11)	15.5 (0.11)
Total carbohydrate (g/d)	239 (1.52)	233.8 (1.5)	235.8 (1.5)	247.1 (1.53)	244.1 (1.5)	236.3 (1.5)	233.7 (1.5)	241.5 (1.5)
CQI score	15.0 (0.09)	12.8 (0.09)	11.4 (0.09)	9.0 (0.09)	14.0 (0.11)	12.4 (0.11)	11.4 (0.11)	10.4 (0.11)
Total fiber (g/1000kcal)	13.4 (0.08)	10.3 (0.07)	8.8 (0.07)	6.9 (0.08)	11.7 (0.10)	10.0 (0.10)	9.1 (0.10)	8.5 (0.10)
Whole grain (svg/d)	1.5 (0.03)	1.1 (0.03)	0.9 (0.03)	0.5 (0.03)	1.8 (0.03)	1.1 (0.03)	0.7 (0.03)	0.4 (0.03)
Refined grain (svg/d)	2.7 (0.05)	3.2 (0.05)	3.3 (0.05)	3.3 (0.05)	3.2 (0.05)	3.5 (0.05)	3.3 (0.05)	2.5 (0.05)
Fruit (svg/d)	2.7 (0.05)	2.2 (0.05)	2.0 (0.05)	1.6 (0.05)	2.2 (0.05)	2.1 (0.05)	2.1 (0.05)	2.2 (0.05)
Vegetables (svg/d)	4.5 (0.05)	3.1 (0.05)	2.4 (0.05)	1.7 (0.05)	3.1 (0.06)	3.0 (0.06)	2.9 (0.06)	2.7 (0.06)
SSBs (svg/d)	0.8 (0.04)	1.0 (0.04)	1.2 (0.04)	1.9 (0.04)	0.9 (0.04)	1.1 (0.04)	1.3 (0.04)	1.7 (0.04)
Total alcohol (g/d)	11.7 (0.56)	11.7 (0.56)	11.2 (0.55)	9.1 (0.57)	10.2 (0.55)	9.6 (0.55)	10.3 (0.55)	13.7 (0.55)
SFA (% kcal/d)	9.5 (0.10)	10.4 (0.10)	10.8 (0.10)	11.0 (0.10)	9.6 (0.10)	10.3 (0.10)	10.8 (0.10)	10.7 (0.10)

Online Supporting Material

Table S2 Continued

	Baseline total fiber				Baseline cereal fiber			
	Q1<br>3.0-12.5	Q2<br>12.5-17.0	Q3<br>17.0-22.2	Q4<br>22.3-72.3	Q1<br>0.2-3.0	Q2<br>3.0-4.6	Q3<br>4.-6.8	Q4<br>6.8-53.6
Characteristics[1]								
n	774	776	775	776	775	775	776	775
Age	54.1 (0.34)	54.4 (0.34)	55.1 (0.34)	56.0 (0.34)	55.4 (0.34)	54.5 (0.34)	54.6 (0.34)	55.1 (0.34)
Sex (%M)	46.9	44.0	46.8	45.2	45.4	43.9	45.7	48.0
Weight (kg)	76.9 (0.5)	77.0 (0.5)	77.0 (0.5)	76.7 (0.5)	76.8 (0.5)	77.6 (0.5)	77.1 (0.5)	76.2 (0.5)
WC (cm)	93.2 (0.45)	92.4 (0.45)	92.8 (0.45)	92.0 (0.45)	93.0 (0.45)	93.4 (0.45)	92.4 (0.45)	91.7 (0.45)
BMI (kg/m^2)	27.4 (0.17)	27.2 (0.17)	27.2 (0.17)	27.0 (0.17)	27.4 (0.17)	27.5 (0.17)	27.2 (0.17)	26.7 (0.17)
PAI score	34.5 (0.22)	35.2 (0.22)	35.0 (0.22)	35.2 (0.22)	35.0 (0.22)	35.1 (0.22)	34.9 (0.22)	34.9 (0.22)
Current smoker (%)	28.0	19.5	14.7	9.9	29.2	19.9	15.4	7.6
Hypertension (%)	45.1	48.3	45.5	47.7	46.9	47.5	47.1	45.1
BP medication (%)	17.3	18.3	18.1	17.4	18.0	17.7	17.5	18.0
Lipid medication (%)	9.0	6.4	6.8	8.3	6.9	8.5	6.2	8.8
Menopausal (%)	35.3	35.4	37.1	36.8	35.3	35.2	36.5	37.6
Dietary Intakes[2]								
Total energy (kcal/d)	1353 (16.6)	1717 (16.5)	2003 (16.5)	2407 (16.5)	1466 (18.8)	1761 (18.8)	1988 (18.8)	2266 (18.8)
Carbohydrate (% E)	47.3 (0.3)	50.1 (0.3)	50.8 (0.3)	55.6 (0.3)	47.6 (0.3)	49.6 (0.3)	51.8 (0.3)	54.8 (0.3)
Fat (% E)	31.6 (0.22)	30.3 (0.22)	30.3 (0.22)	27.5 (0.22)	31.2 (0.22)	31 (0.22)	29.8 (0.22)	27.7 (0.22)
Protein (% E)	16.9 (0.12)	16.7 (0.12)	17.0 (0.12)	16.7 (0.12)	16.7 (0.12)	16.8 (0.12)	16.7 (0.12)	17.0 (0.12)
Total carbohydrate (g/d)	217.7 (1.61)	230.0 (1.42)	237.0 (1.41)	270.9 (1.62)	223.6 (1.54)	230.8 (1.45)	240.6 (1.45)	260.6 (1.54)
CQI score	8.6 (0.11)	10.9 (0.09)	13.2 (0.09)	15.6 (0.11)	10.1 (0.12)	11.3 (0.11)	12.6 (0.11)	14.2 (0.11)
Total fiber (g/1000kcal)	5.6 (0.07)	8.5 (0.06)	10.7 (0.06)	14.6 (0.07)	7.7 (0.10)	9.0 (0.10)	10.2 (0.1)	12.4 (0.10)
Whole grain (svg/d)	0.4 (0.04)	0.8 (0.03)	1.1 (0.03)	1.8 (0.04)	0.3 (0.03)	0.6 (0.03)	1.2 (0.03)	1.9 (0.03)
Refined grain (svg/d)	3.1 (0.06)	3.1 (0.05)	3.1 (0.05)	3.1 (0.06)	2.5 (0.06)	3.1 (0.05)	3.4 (0.05)	3.4 (0.06)
Fruit (svg/d)	1.1 (0.05)	1.7 (0.05)	2.2 (0.05)	3.5 (0.05)	1.9 (0.05)	1.9 (0.05)	2.3 (0.05)	2.5 (0.05)
Vegetables (svg/d)	1.5 (0.06)	2.4 (0.05)	3.2 (0.05)	4.7 (0.06)	2.6 (0.07)	2.8 (0.06)	3.0 (0.06)	3.3 (0.07)
SSBs (svg/d)	1.5 (0.05)	1.3 (0.04)	1.1 (0.04)	1.0 (0.05)	1.4 (0.04)	1.3 (0.04)	1.2 (0.04)	1.0 (0.04)
Total alcohol (g/d)	69.0 (0.54)	64.8 (0.47)	63.2 (0.47)	52.9 (0.54)	66.3 (0.51)	65.5 (0.48)	62.4 (0.48)	55.6 (0.51)
SFA (% kcal/d)	12.2 (0.11)	10.9 (0.09)	10.2 (0.09)	8.3 (0.11)	11.6 (0.10)	10.9 (0.10)	10.1 (0.10)	9.0 (0.10)

Online Supporting Material

Table S2 Continued

	Baseline fruit fiber				Baseline vegetable fiber			
	Q1<br>0-1.4	Q2<br>1.4-2.8	Q3<br>2.8-4.8	Q4<br>4.8-33.6	Q1<br>0.1-2.6	Q2<br>2.6-3.8	Q3<br>3.8-5.5	Q4<br>5.5-39.5
Characteristics[1]								
n	775	775	776	775	775	775	776	775
Age	52.8 (0.34)	54.6 (0.34)	55.1 (0.34)	57.2 (0.34)	54.5 (0.35)	54.7 (0.34)	54.7 (0.34)	55.8 (0.34)
Sex (%M)	52.1	45.2	42.9	42.7	58.0	46.2	41.3	37.4
Weight (kg)	76.9 (0.5)	77.5 (0.5)	76.9 (0.5)	76.3 (0.5)	76.0 (0.5)	77.3 (0.5)	77.0 (0.5)	77.3 (0.5)
WC (cm)	93.5 (0.46)	93.0 (0.45)	92.6 (0.45)	91.4 (0.46)	92.2 (0.46)	93.1 (0.45)	92.4 (0.45)	92.8 (0.46)
BMI (kg/m^2)	27.3 (0.17)	27.3 (0.17)	27.1 (0.17)	27.0 (0.17)	27.0 (0.17)	27.4 (0.17)	27.1 (0.17)	27.2 (0.17)
PAI score	34.8 (0.22)	34.7 (0.22)	35.1 (0.22)	35.3 (0.23)	34.6 (0.22)	34.7 (0.22)	35.1 (0.22)	35.5 (0.23)
Current smoker (%)	31.8	18.7	11.8	9.8	25.1	17.7	15.8	13.6
Hypertension (%)	48.9	46.5	44.9	46.3	45.8	48.0	43.5	49.2
BP medication (%)	17.4	16.6	17.5	19.6	16.1	19.2	19.8	16.0
Lipid medication (%)	6.1	7.7	8.2	8.5	9.0	6.7	7.1	7.7
Menopausal (%)	37.3	35.2	36.0	36.1	35.4	36.9	35.1	37.2
Dietary Intakes[2]								
Total energy (kcal/d)	1656 (20.8)	1808 (20.7)	1881 (20.7)	2136 (20.8)	1560 (20.2)	1803 (20)	1930 (20)	2189 (20.1)
Carbohydrate (% E)	46.5 (0.3)	49.5 (0.3)	51.9 (0.3)	55.9 (0.3)	49.4 (0.3)	50.6 (0.3)	51.3 (0.3)	52.5 (0.3)
Fat (% E)	32.6 (0.22)	30.8 (0.22)	29.3 (0.22)	27 (0.22)	31.4 (0.23)	30.2 (0.22)	29.7 (0.22)	28.4 (0.23)
Protein (% E)	16.5 (0.12)	17.0 (0.12)	17.1 (0.12)	16.7 (0.12)	16.0 (0.12)	16.6 (0.12)	17.0 (0.12)	17.6 (0.12)
Total carbohydrate (g/d)	219.6 (1.43)	229.8 (1.40)	242.1 (1.40)	264.0 (1.44)	233.5 (1.57)	236.9 (1.5)	240.1 (1.5)	245.2 (1.57)
CQI score	10.2 (0.11)	11.4 (0.11)	12.4 (0.11)	14.2 (0.11)	10.2 (0.11)	11.3 (0.11)	12.6 (0.11)	14.2 (0.11)
Total fiber (g/1000kcal)	7.5 (0.09)	8.9 (0.09)	10.3 (0.09)	12.7 (0.09)	7.4 (0.09)	9.0 (0.09)	10.4 (0.09)	12.6 (0.09)
Whole grain (svg/d)	0.7 (0.04)	0.9 (0.03)	1.1 (0.03)	1.3 (0.04)	0.8 (0.04)	0.9 (0.03)	1.1 (0.03)	1.3 (0.04)
Refined grain (svg/d)	3.3 (0.05)	3.2 (0.05)	3.1 (0.05)	2.9 (0.05)	3.2 (0.06)	3.3 (0.05)	3.1 (0.05)	3.0 (0.06)
Fruit (svg/d)	0.8 (0.04)	1.7 (0.04)	2.3 (0.04)	3.8 (0.04)	1.6 (0.05)	2.0 (0.05)	2.3 (0.05)	2.7 (0.05)
Vegetables (svg/d)	2.3 (0.06)	2.6 (0.06)	3.0 (0.06)	3.8 (0.06)	1.3 (0.04)	2.2 (0.04)	3.1 (0.04)	5.2 (0.04)
SSBs (svg/d)	1.1 (0.04)	1.3 (0.04)	1.3 (0.04)	1.2 (0.04)	1.3 (0.04)	1.3 (0.04)	1.2 (0.04)	1.1 (0.04)
Total alcohol (g/d)	14.7 (0.56)	11.8 (0.55)	9.6 (0.55)	7.6 (0.57)	11.7 (0.58)	11.4 (0.56)	10.4 (0.56)	10.3 (0.58)
SFA (% kcal/d)	11.8 (0.10)	10.8 (0.10)	10 (0.09)	9.0 (0.10)	11.5 (0.10)	10.6 (0.10)	10.1 (0.10)	9.3 (0.10)

Online Supporting Material

Table S2 Continued

	Baseline glycemic index				Baseline solid to total carbohydrate ratio			
	Q1<br>36.0-52.3	Q2<br>52.4-54.8	Q3<br>54.8-56.9	Q4<br>56.9-69.4	Q1<br>0.1-0.8	Q2<br>0.8-0.9	Q3<br>0.9-0.9	Q4<br>0.9-1.0
Characteristics[1]								
n	773	776	776	776	775	775	776	775
Age	55.1 (0.34)	54.5 (0.34)	55.1 (0.34)	54.9 (0.34)	53.8 (0.34)	55.3 (0.34)	55.3 (0.34)	55.3 (0.35)
Sex (%M)	41.6	45.2	49.6	46.5	56.3	50.5	45.2	30.9
Weight (kg)	76.8 (0.5)	77.0 (0.5)	77.2 (0.5)	76.6 (0.5)	76.3 (0.5)	76.6 (0.5)	76.7 (0.5)	78 (0.5)
WC (cm)	92.7 (0.45)	92.5 (0.45)	92.3 (0.45)	92.9 (0.45)	92.5 (0.46)	91.9 (0.45)	92.3 (0.45)	93.8 (0.46)
BMI (kg/m^2)	27.2 (0.17)	27.1 (0.17)	27.2 (0.17)	27.2 (0.17)	27.1 (0.17)	26.8 (0.17)	27.1 (0.17)	27.7 (0.17)
PAI score	35.3 (0.23)	34.9 (0.22)	35 (0.22)	34.5 (0.22)	35.5 (0.22)	34.9 (0.22)	34.6 (0.22)	34.8 (0.23)
Current smoker (%)	19.4	17.3	15.2	20.2	23.2	16.1	16.4	16.4
Hypertension (%)	44.4	43.0	49.5	49.6	46.9	46.9	47.3	45.5
BP medication (%)	17.0	17.8	16.7	19.6	17.3	18.9	17.9	17.0
Lipid medication (%)	6.6	7.1	9.5	7.3	9.9	6.6	6.8	7.2
Menopausal (%)	36.9	36.2	34.2	37.3	37.4	35.1	35.7	36.4
Dietary Intakes[2]								
Total energy (kcal/d)	1768 (21.5)	1867 (21.4)	1893 (21.4)	1952 (21.4)	1938 (21.6)	1866 (21.4)	1920 (21.4)	1757 (21.7)
Carbohydrate (% E)	47.9 (0.3)	50.0 (0.3)	51.7 (0.3)	54.2 (0.3)	54.2 (0.3)	50.4 (0.3)	50.6 (0.3)	48.6 (0.3)
Fat (% E)	30.1 (0.23)	30.5 (0.23)	30.0 (0.23)	29.1 (0.23)	28.4 (0.23)	30.0 (0.23)	30.3 (0.23)	31.0 (0.23)
Protein (% E)	17.7 (0.11)	17.2 (0.11)	16.6 (0.11)	15.7 (0.11)	15.3 (0.11)	16.9 (0.11)	17.2 (0.11)	17.9 (0.11)
Total carbohydrate (g/d)	224.9 (1.46)	234.3 (1.45)	241.1 (1.45)	255.3 (1.46)	253.7 (1.49)	235.7 (1.47)	236.7 (1.47)	229.7 (1.5)
CQI score	14.4 (0.10)	12.9 (0.10)	11.5 (0.1)	9.5 (0.1)	9.0 (0.09)	11.5 (0.09)	13.2 (0.09)	14.6 (0.09)
Total fiber (g/1000kcal)	10.1 (0.11)	10 (0.11)	10.0 (0.11)	9.3 (0.11)	8.5 (0.11)	9.8 (0.11)	10.3 (0.11)	10.7 (0.11)
Whole grain (svg/d)	0.9 (0.04)	1.0 (0.03)	1.1 (0.03)	1.0 (0.04)	0.8 (0.03)	1.0 (0.03)	1.1 (0.03)	1.2 (0.04)
Refined grain (svg/d)	2.5 (0.05)	3.0 (0.05)	3.3 (0.05)	3.6 (0.05)	2.9 (0.05)	3.1 (0.05)	3.3 (0.05)	3.2 (0.05)
Fruit (svg/d)	2.2 (0.05)	2.3 (0.05)	2.2 (0.05)	1.9 (0.05)	2.5 (0.05)	2.3 (0.05)	2.1 (0.05)	1.7 (0.05)
Vegetables (svg/d)	3.4 (0.06)	3.1 (0.06)	2.9 (0.06)	2.4 (0.06)	2.6 (0.06)	2.9 (0.06)	3.0 (0.06)	3.2 (0.06)
SSBs (svg/d)	0.9 (0.04)	1.1 (0.04)	1.3 (0.04)	1.6 (0.04)	2.6 (0.03)	1.3 (0.03)	0.8 (0.03)	0.2 (0.03)
Total alcohol (g/d)	16.4 (0.55)	11.1 (0.54)	9.5 (0.54)	6.7 (0.54)	10.3 (0.56)	12.1 (0.56)	10.1 (0.56)	11.2 (0.56)
SFA (% kcal/d)	10.7 (0.10)	10.6 (0.10)	10.3 (0.10)	9.9 (0.10)	9.9 (0.10)	10.5 (0.10)	10.4 (0.10)	10.7 (0.10)

Online Supporting Material

Table S2 Continued

	Baseline whole grain to total grain ratio			
	Q1<br>0-0.1	Q2<br>0.1-0.2	Q3<br>0.2-0.3	Q4<br>0.3-1.0
Characteristics[1]				
n	775	775	776	775
Age	55.5 (0.34)	53.5 (0.34)	54.1 (0.34)	56.5 (0.34)
Sex (%M)	48.3	47.7	44.6	42.4
Weight (kg)	77.3 (0.5)	77.4 (0.5)	76.8 (0.5)	76.1 (0.5)
WC (cm)	93.1 (0.45)	93.1 (0.45)	92.2 (0.45)	92.1 (0.45)
BMI (kg/m^2)	27.5 (0.17)	27.3 (0.17)	27.0 (0.17)	26.8 (0.17)
PAI score	35.1 (0.22)	35.1 (0.22)	34.9 (0.22)	34.8 (0.22)
Current smoker (%)	24.0	21.5	14.9	11.7
Hypertension (%)	50.5	47.0	43.9	45.1
BP medication (%)	17.2	19.4	16.6	17.9
Lipid medication (%)	6.8	6.3	8.5	8.8
Menopausal (%)	35.8	37.7	35.4	35.7
Dietary Intakes[2]				
Total energy (kcal/d)	1802 (21.5)	1933 (21.5)	1905 (21.5)	1841 (21.6)
Carbohydrate (% E)	49.6 (0.3)	50.0 (0.3)	51.3 (0.3)	53.0 (0.3)
Fat (% E)	30.9 (0.22)	31.0 (0.22)	29.7 (0.22)	28.2 (0.23)
Protein (% E)	16.1 (0.12)	16.7 (0.12)	17.0 (0.12)	17.5 (0.12)
Total carbohydrate (g/d)	233.6 (1.49)	233.4 (1.50)	240.1 (1.49)	248.4 (1.50)
CQI score	9.6 (0.10)	11.1 (0.10)	12.9 (0.10)	14.6 (0.10)
Total fiber (g/1000kcal)	8.4 (0.10)	9.1 (0.10)	10.2 (0.10)	11.7 (0.10)
Whole grain (svg/d)	0.2 (0.02)	0.5 (0.02)	1.1 (0.02)	2.1 (0.02)
Refined grain (svg/d)	3.9 (0.05)	3.4 (0.05)	3.0 (0.05)	2.2 (0.05)
Fruit (svg/d)	1.8 (0.05)	1.9 (0.05)	2.3 (0.05)	2.5 (0.05)
Vegetables (svg/d)	2.6 (0.06)	2.7 (0.06)	3.1 (0.06)	3.3 (0.06)
SSBs (svg/d)	1.3 (0.04)	1.2 (0.04)	1.2 (0.04)	1.2 (0.04)
Total alcohol (g/d)	13 (0.56)	10.7 (0.56)	10.5 (0.55)	9.5 (0.56)
SFA (% kcal/d)	10.9 (0.10)	10.8 (0.10)	10.3 (0.10)	9.6 (0.10)

[1]Adjusted for age and sex

[2]Adjusted for age, sex, and total energy intake

BMI (body mass index); CQI (carbohydrate quality index); E (total energy intake); PAI (physical activity index); SFA (saturated fatty acid); SSB (sugar-sweetened beverage); WC (waist circumference)

Online Supporting Material

Table S3. Means (SE) of four-year change in waist circumference (cm) by quartiles of components of the CQI in 3101 participants of the Framingham Cohort Study

	Quartiles				
	1	2	3	4	*P*-trend
Glycemic index					
n (observations)	2262	2262	2264	2265	
Median (range)	50.3 (28.1-51.9)	53.1 (51.9-54.0)	54.9 (54.0-55.9)	57.2 (55.9-64.8)	
Model 1	1.72 (0.10)	2.03 (0.10)	2.39 (0.10)	2.49 (0.11)	<0.001
Model 2	1.74 (0.10)	2.03 (0.10)	2.40 (0.11)	2.52 (0.11)	<0.001
Model 3	1.85 (0.11)	2.18 (0.11)	2.57 (0.11)	2.86 (0.12)	<0.001
Total fiber					
n	2261	2266	2263	2263	
Median (range)	11.4 (3.1-13.9)	16.1 (13.9-18.0)	20.3 (18.1-23.1)	27.2 (23.1-78.6)	
Model 1	2.45 (0.12)	2.27 (0.11)	2.11 (0.10)	1.71 (0.12)	<0.001
Model 2	2.54 (0.13)	2.29 (0.11)	2.11 (0.10)	1.68 (0.13)	<0.001
Model 3	2.78 (0.14)	2.50 (0.11)	2.26 (0.11)	1.83 (0.14)	<0.001
Whole grain: total grain					
n	2263	2263	2264	2263	
Median (range)	0.09 (0-0.16)	0.22 (0.16-0.28)	0.35 (0.28-0.42)	0.53 (0.42-1.00)	
Model 1	2.98 (0.12)	2.42 (0.11)	2.03 (0.10)	1.52 (0.09)	<0.001
Model 2	3.06 (0.12)	2.45 (0.11)	2.04 (0.10)	1.51 (0.09)	<0.001
Model 3	3.15 (0.13)	2.59 (0.11)	2.24 (0.10)	1.70 (0.10)	<0.001
Solid: total carbohydrate					
n	2263	2263	2264	2263	
Median (range)	0.77 (0.25-0.83)	0.86 (0.83-0.89)	0.92 (0.89-0.95)	0.98 (0.95-1.00)	
Model 1	2.14 (0.10)	2.24 (0.10)	2.09 (0.10)	2.05 (0.10)	0.380
Model 2	2.17 (0.11)	2.25 (0.10)	2.13 (0.10)	2.04 (0.10)	0.307
Model 3	2.44 (0.11)	2.46 (0.11)	2.24 (0.11)	2.21 (0.11)	0.084

Model 1: periodic baseline age, sex, energy, periodic baseline waist circumference
Model 2: Model 1 + current smoker (y/n), physical activity score, alcohol (g/d), menopausal status, medication use for diabetes, SFA (% kcal/d)
Model 3: Model 2 + periodic baseline BMI
BMI (body mass index); CQI (carbohydrate quality index); SFA (saturated fatty acid)

Online Supporting Material

Table S4. Means (SE) of four-year change in body weight (kg) by quartiles of different carbohydrate quality metrics in 3101 participants of the Framingham Cohort Study

	Energy Adjusted Quartiles				
	1	2	3	4	*P*-trend
CQI					
n (observations)	2263	2263	2264	2263	
Median (range)	8.5 (3.7-9.9)	11.0 (9.9-12.0)	13.0 (12.0-14.0)	15.5 (14.0-21.2)	
Model 1	0.22 (0.08)	0.52 (0.09)	0.49 (0.09)	0.29 (0.08)	0.643
Model 2	0.23 (0.09)	0.53 (0.09)	0.48 (0.09)	0.31 (0.09)	0.604
Model 3	0.23 (0.09)	0.54 (0.09)	0.47 (0.09)	0.29 (0.09)	0.737
Total carbohydrate					
n	2263	2263	2264	2263	
Median (range)	180 (36-197)	210 (197-220)	230 (220-242)	258 (242-366)	
Model 1	0.38 (0.08)	0.37 (0.08)	0.39 (0.09)	0.38 (0.09)	0.988
Model 2	0.38 (0.11)	0.36 (0.09)	0.40 (0.09)	0.40 (0.11)	0.898
Model 3	0.39 (0.11)	0.36 (0.09)	0.39 (0.09)	0.39 (0.11)	0.991
Total fiber					
n	2263	2263	2264	2263	
Median (range)	12.8 (5.6-14.7)	16.2 (14.7-17.6)	19.2 (17.6-21.1)	23.9 (21.1-54.1)	
Model 1	0.41 (0.09)	0.41 (0.09)	0.41 (0.09)	0.29 (0.09)	0.290
Model 2	0.44 (0.10)	0.42 (0.09)	0.42 (0.09)	0.27 (0.09)	0.232
Model 3	0.45 (0.10)	0.41 (0.09)	0.42 (0.09)	0.26 (0.09)	0.194
Cereal fiber					
n	2263	2263	2264	2263	
Median (range)	3.0 (0.4-3.8)	4.4 (3.8-5.0)	5.7 (5.0-6.7)	8.0 (6.7-33.8)	
Model 1	0.32 (0.09)	0.47 (0.09)	0.44 (0.09)	0.29 (0.08)	0.565
Model 2	0.35 (0.09)	0.47 (0.09)	0.44 (0.09)	0.29 (0.09)	0.457
Model 3	0.36 (0.09)	0.47 (0.09)	0.43 (0.09)	0.28 (0.09)	0.368
Vegetable fiber					
n	2263	2263	2264	2263	
Median (range)	2.3 (0.0-2.9)	3.5 (2.9-4.0)	4.6 (4.0-5.4)	6.7 (5.4-27.7)	
Model 1	0.36 (0.08)	0.35 (0.09)	0.39 (0.09)	0.42 (0.08)	0.512
Model 2	0.32 (0.09)	0.36 (0.09)	0.41 (0.09)	0.46 (0.09)	0.235
Model 3	0.31 (0.09)	0.35 (0.09)	0.42 (0.09)	0.46 (0.09)	0.209
Fruit fiber					
n	2258	2259	2259	2258	
Median (range)	1.2 (0.0-1.9)	2.6 (1.9-3.3)	4.0 (3.3-4.9)	6.3 (4.9-24.7)	
Model 1	0.48 (0.08)	0.44 (0.09)	0.32 (0.09)	0.28 (0.09)	0.066
Model 2	0.51 (0.09)	0.45 (0.09)	0.31 (0.09)	0.26 (0.09)	0.054
Model 3	0.51 (0.09)	0.46 (0.09)	0.31 (0.09)	0.25 (0.09)	0.044
Carbohydrate: total fiber					
n	2263	2263	2264	2263	
Median (range)	9.6 (4.2-10.6)	11.5 (10.6-12.3)	13.3 (12.3-14.5)	16.5 (14.5-55.0)	
Model 1	0.38 (0.08)	0.54 (0.09)	0.31 (0.09)	0.29 (0.09)	0.191

Model 2	0.39 (0.08)	0.55 (0.09)	0.29 (0.09)	0.30 (0.09)	0.196
Model 3	0.39 (0.08)	0.55 (0.09)	0.28 (0.09)	0.31 (0.09)	0.218
Carbohydrate: cereal fiber					
n	2263	2263	2264	2263	
Median (range)	29.7 (9.2-35.3)	40.4 (35.3-45.6)	51.6 (45.6-60.0)	75.3 (60.0-7885.1)	
Model 1	0.39 (0.08)	0.38 (0.09)	0.47 (0.09)	0.27 (0.09)	0.370
Model 2	0.39 (0.08)	0.38 (0.09)	0.48 (0.09)	0.29 (0.09)	0.447
Model 3	0.38 (0.08)	0.38 (0.09)	0.48 (0.09)	0.29 (0.09)	0.530

Model 1: periodic baseline age, sex, energy, periodic baseline waist circumference
Model 2: Model 1 + current smoker (y/n), physical activity score, alcohol (g/d), menopausal status, medication use for diabetes, SFA (% kcal); models for subtypes of fiber are mutually adjusted for the other two subtypes of fiber
Model 3: Model 2 + periodic baseline BMI
BMI (body mass index); CQI (carbohydrate quality index); SFA (saturated fatty acid)
